# Features of Cytokine Storm Identified by Distinguishing Clinical Manifestations in COVID-19

**DOI:** 10.3389/fpubh.2021.671788

**Published:** 2021-05-24

**Authors:** Wei-Xi Shen, Rong-Cheng Luo, Jing-Quan Wang, Zhe-Sheng Chen

**Affiliations:** ^1^Shenzhen Hospital, Southern Medical University, Shenzhen, China; ^2^Shenzhen Tianyou Medical Institute, Shenzhen, China; ^3^College of Pharmacy and Health Science, St. John's University, New York, NY, United States

**Keywords:** coronavirus disease 2019 (COVID-19), clinical manifestation, cytokine storm, pathogenesis, transforming growth factor-β (TGF-β)

## Abstract

Coronavirus disease 2019 (COVID-19) is caused by a new coronavirus, namely severe acute respiratory syndrome coronavirus 2 (SARS-CoV-2) and is currently spreading all over the world. In this paper, we developed a practical model for identifying the features of cytokine storm, which is common in acute infectious diseases and harmful manifestation of COVID-19, by distinguishing major and minor clinical events. This model is particularly suitable for identifying febrile and infectious diseases like COVID-19. Based on this model, features of cytokine storm and pathogenesis of COVID-19 have been proposed to be a consequence of the disequilibrated cytokine network resulting from increased biological activity of transforming growth factor-β (TGF-β), which induces certain clinical manifestations such as fatigue, fever, dry cough, pneumonia, abatement and losing of olfactory, and taste senses in some patients. Research and clarification of the pathogenesis of COVID-19 will contribute to precision treatment. Various anti-TGF-β therapies may be explored as potential COVID-19 treatment. This novel model will be helpful in reducing the widespread mortality of COVID-19.

## Introduction

The coronavirus disease 2019 (COVID-19) pandemic is currently spreading worldwide and contributing to widespread mortality. There is an urgent need to clarify the pathogenic mechanism underlying the severe acute respiratory syndrome coronavirus 2 (SARS-CoV-2) infection. In response, on the basis of our comprehensive clinical experience, profound understanding of cytokine network, and the recognition of the comparative research between Chinese medicine and modern medicine over the past years (1–4), we developed a practical diagnostic model to judge and identify the features of cytokine storm of acute infectious diseases by distinguishing the clinical manifestations of patients. The features of cytokine storm elicited by COVID-19 were clarified as a typical application of this model.

## Clinical Manifestations of COVID-19

The main symptoms of COVID-19 are fever, fatigue, dry cough, abatement and losing of olfactory and taste senses. Patients with severe symptoms may also experience dyspnea, hypoxemia, shock, and multiple organ failure (5, 6). In the early stage of COVID-19, patients usually have unchanged or decreased number of white blood and decreased number of lymphocytes, with multiple small patch shadows and interstitial lesions on lung CT images. Autopsies on patients with COVID-19 show obvious inflammation of lungs accompanied with large amount of mucus (7).

The clinical manifestations of COVID-19 can be divided into four categories. (1) The major manifestation: A major symptom of COVID-19 is fatigue, with the degree of fatigue being associated with the clinical type of COVID-19. (2) Minor manifestations: fever, dry cough, abatement and losing of olfactory and taste senses, interstitial lung changes, and lymphopenia. The major and minor manifestations are directly caused by the disequilibrated cytokines of COVID-19. (3) The manifestations due to secondary pathogenic processes: sputum and chest distress caused by secondary lung bacterial infection. Patients with severe symptoms may display respiratory failure, shock, and multiple organ failure, as well as blood pressure reduction, poor peripheral perfusion, and so on. (4) The manifestations of comorbidities: comorbidities such as diabetes, hypertension, and coronary heart disease may be enhanced by the infection and progression of COVID-19 (8). The mixed manifestations make the identification of cytokine storm and actual pathogenesis of COVID-19 extremely difficult.

## A Practical Diagnostic Model to Identify Cytokines and Clinical Manifestations

The understanding and recognition of diseases are achieved based on the systematic investigation of etiology, pathogenesis, pathogenic process, clinical manifestations, treatment, and rehabilitation. The dysfunction of cytokine network and clinical manifestations of diseases are closely associated with each other and have a causal relationship. Different pathogenesis and disequilibrated cytokine network status will yield different pathological processes, and in return, different manifestations.

Cytokines are a group of polypeptides functioning as first messenger molecules, and play key roles during the occurrence, development, and evolution of diseases. Clinical manifestations of diseases depend on the leading cytokines which play dominant roles in the disequilibrated cytokine network status.

Based on the causal relationship between cytokines and manifestations, we developed a practical diagnostic model to identify the features of cytokine storm as a result of acute infectious diseases. This model includes the following diagnostic procedures: (1) observing and distinguishing the patient's comprehensive clinical manifestations; (2) differentiating the major, minor, secondary, and other manifestations; (3) judging the leading cytokines that display the dominant roles of the disequilibrated cytokine network and eliciting the manifestations of diseases, and; (4) identifying the features of cytokine storm of the diseases. Following this model, the pathogenesis and features of cytokine storm of COVID-19 infection was elucidated in the following.

Thus, it can be seen that when we judge and identify the cytokine storm, we are particularly focusing on the initial clinical manifestations of the disease. This is because at this time the manifestations are caused by the genuine cytokine storm of the primary disease without other noisy factors. The clinical manifestations are much more complicated after the disease has triggered a series of secondary pathological processes. Moreover, the basis and key to identify cytokine storm feature still need to have a profound understanding of cytokine network, and master the biological activities of various cytokines and current knowledge of cytokines progress.

## Features of Cytokine Storm and Pathogenesis of COVID-19

Abnormal expression and dysfunction of cytokines in diseases can lead to a certain disequilibrated cytokine network and elicit corresponding pathogenic changes and manifestations (9–11). The disequilibrated cytokine network status of diseases can be divided into three levels: mild, ordinary, and critical. Commonly, a critical level of disequilibrated cytokine network status is known as cytokine storm, which means an excessive number and quantity of cytokines are released. Cytokine storm can lead to the production of a number of biological mediators that interfere with normal cell function and cause systematic alterations, causing inflammation in the area of the body being flooded, which can be fatal.

Increasing evidence has shown that a number of cytokines, including interleukins (IL-1, IL-6, IL-8), tumor necrosis factor (TNF), a-nd interferon (IFN) displayed significant changes in COVID-19 patients (12). However, the features of cytokine storm of COVID-19 have not been well-identified due to the complexity of the cytokine network. And few studies have focused on the change and role of transforming growth factor-β (TGF-β) during COVID-19 infection.

**Figure 1:**
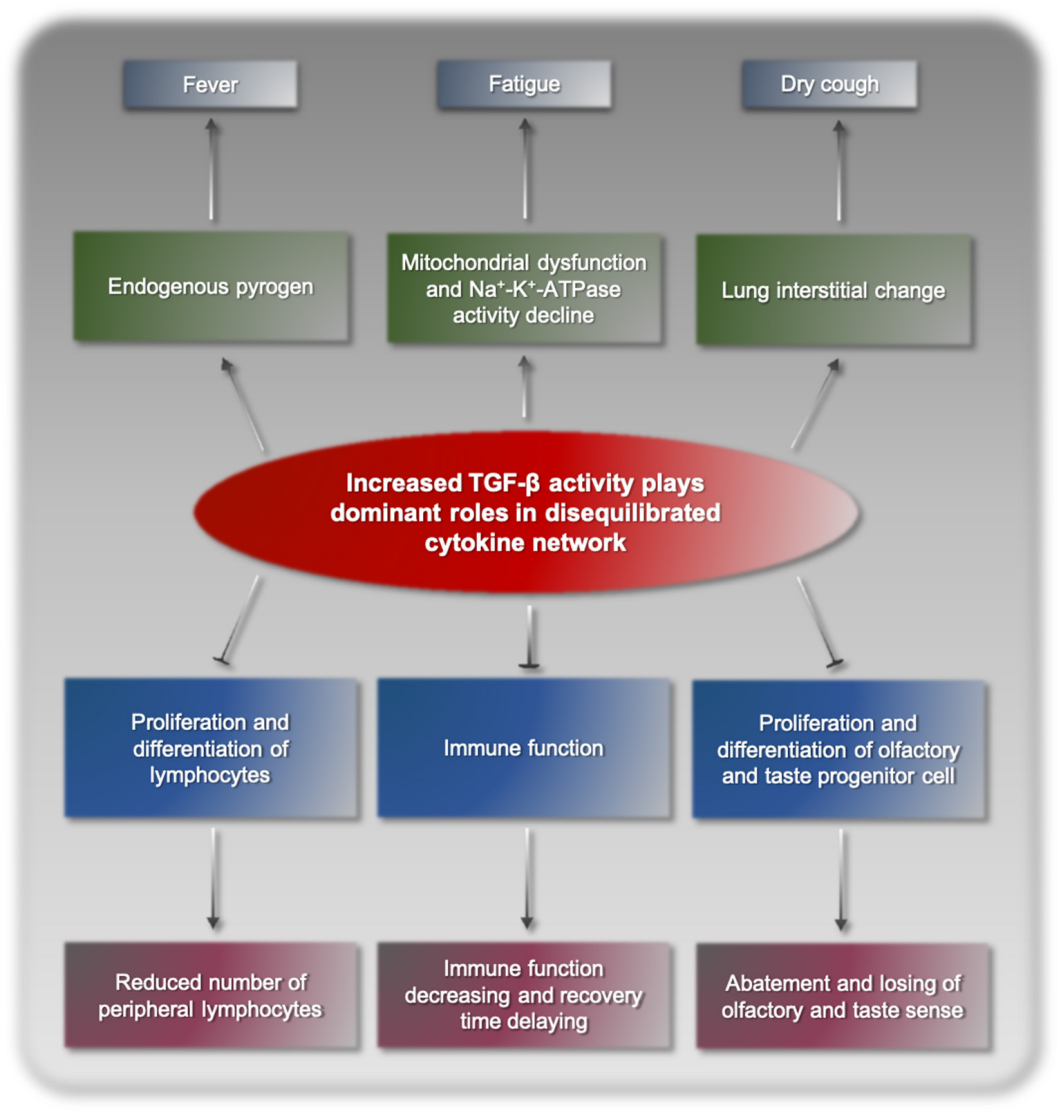
The pathogenic mechanism of COVID-19 manifestations.

Following the above model, we proposed that cytokine storm in COVID-19 was a consequence of disequilibrated cytokine network resulting from increased biological activity of TGF-β. SARS-CoV-2 viruses proliferate and reproduce in their target cells, inducing abnormal stress and immune responses of the body. This process disturbs the normal expression and functional regulation mechanisms of cytokines, finally resulting in disequilibrated cytokine network dominated by TGF-β and its synergetic factors. TGF-β binds to its corresponding receptors and triggers a series of cascade biochemical reactions, pathological processes, and clinical manifestations (Figure 1). TGF-β is a cytokine superfamily and has a broad spectrum of biological activities in the body. As an endogenous pyrogen, TGF-β can cause fever, but its pyrogenicity is relatively weak and usually only results in low fever (13). Fatigue is a typical symptom of COVID-19, potentially due to mitochondrial dysfunction and decline Na^+^-K^+^-ATPase activity (14, 15). Dry cough and interstitial lung change are also caused by increased TGF-β, which is a fibroblast growth factor and has been shown to induce interstitial lung change. TGF-β promotes the secretion of bronchial mucus cells, induces large amounts of thick mucus and pulmonary sputum embolism in the lungs, which hinders normal breathing, and may lead to critical complications, such as serious infections and shock. In addition, TGF-β is a strong immunosuppressive factor in the body, significantly suppressing immune function and delaying recovery (16). Furthermore, TGF-β can inhibit the proliferation and differentiation of lymphocytes (17), reducing the number of peripheral blood lymphocytes. TGF-β has negative impacts on olfactory and gustatory receptor neurons and neurogenesis by inhibiting progenitor cell proliferation and differentiation, elicits the abatement and losing of olfactory and gustatory sense in some patients (18–20). Overall, the clinical manifestations of COVID-19 are highly consistent with increased TGF-β activity, which is the basic evidence supporting TGF-β as the main player of cytokine storm resulting from SARS-CoV-2 infection.

## Heterogeneity and Discrepancy of COVID-19 Cytokine Storm

**Figure 2:**
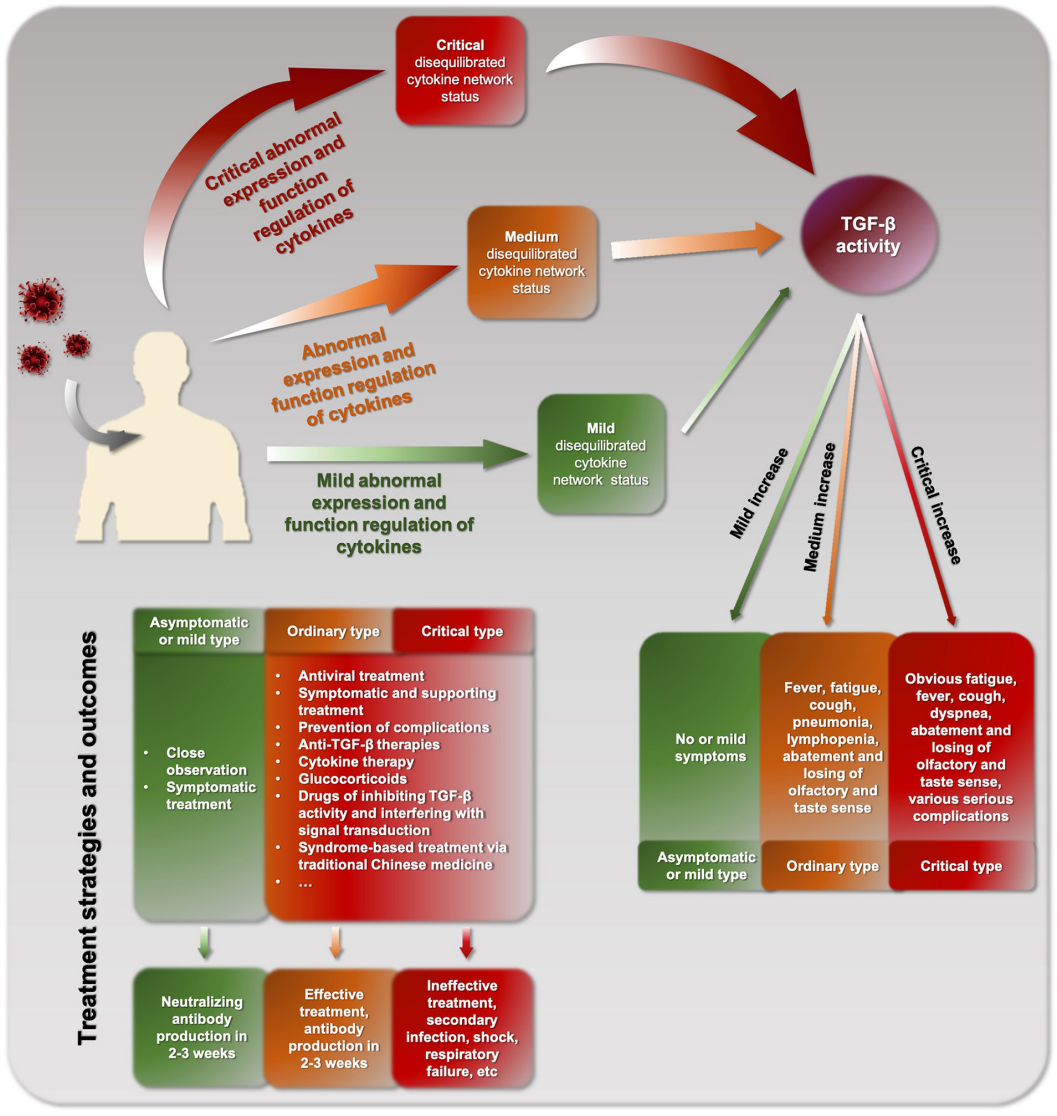
The overall pathogenic process and cytokine storm of COVID-19.

COVID-19 patients can be divided into three types: asymptomatic/mild, ordinary, or critical types (Figure 2), which are consistent with the corresponding levels of mild, moderate, and critical cytokine storm, respectively. The different clinical types might be due to different cytokine storm and different immune response of the body. A basic feature of COVID-19 cytokine storm is an abnormally increased TGF-β activity. However, due to the differences between individuals, different COVID-19 patients and even the same patient at different stages may show inconsistent pathogenesis and clinical manifestations. In clinical practice, physicians should strive to make a timely identification based on such clinical manifestations. For example, when the disease is mainly characterized by obvious fatigue and low fever, TGF-β might be the dominant factor. In contrast, when the main manifestation is hyperpyrexia, IL-1, and TNF-α might be increased and more likely to be the dominant factors.

By comparing the clinical manifestations of COVID-19 and SARS, we see significant difference in cytokine storm. The clinical manifestations of SARS include high fever, cough, dyspnea, and fatigue. The major symptom of high fever of SARS suggests that the leading factors of cytokine storm is related to the increased activity of inflammatory cytokines such as IL-1 and TNF-α. However, the major symptoms of COVID-19 are fatigue and relatively mild fever indicate that it is highly associated with increased activity of TGF-β.

## Significance of Clarifying the Feature of Cytokine Storm

Clarifying the feature of cytokine storm of COVID-19 is helpful for selecting precise treatment and improving efficacy. Currently, there is no antiviral agent that works against SARS-CoV-2, although there is something like Remdesvir and hydroxychloroquine. Hydroxychloroquine has shown some effects when administered with azithromycin in some studies, but more recent study conclude that hydroxychloroquine alone is not effective for the treatment of COVID-19 and that the combination of hydroxychloroquine and azithromycin increases the risk of mortality (21, 22). Although the US FDA granted the emergency use of Remdesivir in April, 2020, and a recent report by Beigel et al. concluded that Remdesivir was superior to placebo in shortening the time to recovery in patients who were hospitalized with COVID-19, Remdesivir does not seem to reduce COVID-19 mortality so far (23). Thus, aside from symptomatic and supportive care, prevention and treatment of complications, various effective anti-TGF-β therapies should be explored, including cytokine therapy, cytokine neutralizing antibodies, glucocorticoids, Chinese herbs, drugs that inhibit TGF-β activity, and interfere with TGF-β signal transduction. Previous studies have supported that targeting TGF-β activity is effective against COVID-19. Some traditional Chinese drugs, such as *Suctellaria baicalensis* and *Utrica dioica* (24), as well as ginseng, have an inhibitory effect on TGF-β activity and can be used to alleviated symptoms of COVID-19 (25). Kaempferol, which can inhibit the secretion of mucus from goblet cells, is beneficial for the prevention and treatment of mucus hypersecretion of the lungs (26, 27). Diterpene phenol extract of *Rosmarinus officinalis* has been reported to inhibit TGF-β in rats (28). N-acetylcysteine (NAC) was used for the prevention and treatment of COVID-19. NAC has mucolytic (anti-mucous) effects. In addition, NAC can inactivate TGF-β and prevent its binding to its receptor (29). Recently, we adopted a strategic approach to repurpose the use of clinically approved drugs against SARS-CoV-2. We uncovered that NAC is able to interrupt the spike protein of the virus and therefore, stop the virus from entering into the host cells. Our results support current clinical trials of NAC for treating severe COVID-19 patients (30).

## Discussion

Great progress has been made in recognizing and detecting the substantial and structural changes of diseases. This is insufficient, however, to comprehensively understand and master the holistic dysfunctional changes of the whole body. The changing regulation and dysfunctional status of the cytokine network make it difficult to determine and identify only by detecting the content changes of a lot of or even all the cytokines. This study proposes a model and reveals the relationship between cytokines and clinical manifestations. The practical diagnostic model we used in identifying the features of cytokine storm could guide diagnosis and treatment of COVID-19 and other infectious diseases.

## Data Availability Statement

The original contributions presented in the study are included in the article/supplementary material, further inquiries can be directed to the corresponding author/s.

## Author Contributions

W-XS, R-CL, and Z-SC conceptualized the manuscript. W-XS wrote the manuscript. R-CL, J-QW, and Z-SC edited the manuscript. All authors read and approved the final version for submission.

## Conflict of Interest

The authors declare that the research was conducted in the absence of any commercial or financial relationships that could be construed as a potential conflict of interest.

## References

[1] Shen WX Sun Y Zhang SR Yu GQ. Study on correlation between IL-1 and other cytokines and essence of lung Yin-deficiency syndrome. J Tradit Chin Med. (2000) 41:423–25.

[2] Shen WX Sun Y Zhang SR. Immunohistochemical research of correlation between IL-1β, IL-6, TNFα, IFNγ in lung cancer cells and lung cancer Yin-deficiency syndrome. Chin J Base Med Tradit Chin Med. (2000) 6:798–801.

[3] Shen WX Sun Y. Investigation of relation between Qi-deficiency syndrome TGF-β1, TNFα PDGF. Chin J Integr Tradit West Med. (2008) 28:950–6.

[4] Shen WX Sun Y. Modern medical principle of diagnosis and treatment of TCM. Med Phil. (2004) 25:55–6.

[5] Guan W Ni Z Hu Y Liang W Qu C He J. Clinical characteristics of coronavirus disease 2019 in China. New Eng J Med. (2020) 382:1708–20. 10.1056/NEJMoa2002032 32109013 PMC7092819

[6] Zachariah P Johnson CL Halabi KC Ahn D. Epidemiology, clinical features, and disease severity in patients with coronavirus disease 2019 (COVID-19) in a children's hospital in New York City, New York. JAMA Pediatr. (2020) 174:e202430. 10.1001/jamapediatrics.2020.2430 32492092 PMC7270880

[7] Yao XH Li TY He ZC Ping YF Liu HW Yu SC. A pathological report of three COVID-19 cases by minimally invasive autopsies. Chin J Pathol. (2020) 49:411–7. 10.3760/cma.j.cn112151-20200312-00193 32172546

[8] Chen Y Gong X Wang L Guo J. Effects of hypertension, diabetes and coronary heart disease on COVID-19 diseases severity: a systematic review and meta-analysis. Medrxiv [Preprint]. (2020). 10.1101/2020.03.25.20043133

[9] Fahy RJ Lichtenberger F McKeegan CB Nuovo GJ Marsh CB Wewers MD. The acute respiratory distress syndrome: a role for transforming growth factor-β1. Am J Respir Cell Mol Biol. (2020) 28:499–503. 10.1165/rcmb.2002-0092OC 12654639

[10] Blobe GC Schiemann WP Lodish HF. Role of transforming growth factor-β in human disease. New Eng J Med. (2000) 342:1350–58. 10.1056/NEJM200005043421807 10793168

[11] Border WA Noble NA. Transforming growth factor β in tissue fibrosis. New Eng J Med. (1994) 331:1286–92. 10.1056/NEJM199411103311907 7935686

[12] Liu Y Zhang C Huang F Yang Y Wang FX Yuan J. Elevated plasma level of selective cytokines in COVID-19 patients reflect viral load and lung injury. Nat Sci Rev. (2020) 7:1003–11. 10.1093/nsr/nwaa037 PMC7107806 34676126

[13] Matsumura S Shibakusa T Fujikawa T Yamada H Fushiki T. Increase in transforming growth factor-β in the brain during infection is related to fever, not depression of spontaneous motor activity. Neuroscience. (2007) 144:1133–40. 10.1016/j.neuroscience.2006.10.037 17156928

[14] Casalena G Bottinger E Daehn I. TGF-β-induced actin cytoskeleton rearrangement in podocytes is associated with compensatory adaptation of mitochondrial energy metabolism. Nephron. (2015) 131:278–84. 10.1159/000442051 26613578 PMC4687741

[15] Pekary AE Levin SR Johnson DG Berg L Hershman JM. Tumor necrosis factor-α (TNF-α) and transforming growth factor-β1 (TGF-β1) inhibit the expression and activity of Na/K-ATPase in FRTL-5 rat thyroid cells. J Interferon Cytokine Res. (1997) 17:185–95. 10.1089/jir.1997.17.185 9142647

[16] Sheng J Chen W Zhu HJ. The immune suppressive function of transforming growth factor-β (TGF-β) in human diseases. Growth Factors. (2015) 33:92–101. 10.3109/08977194.2015.1010645 25714613

[17] Jin B Scott JL Vadas MA Burns GF. TGF beta down-regulates TLiSA1 expression and inhibits the differentiation of precursor lymphocytes into CTL and LAK cells. Immunology. (1989) 66:570–6. 2541074 PMC1385159

[18] Mahanthappa NK Schwarting GA. Peptide growth factor control of olfactory neurogenesis and neuron survival in vitro: roles of EGF and TGF-βs. Neuron. (1993) 10:293–305. 10.1016/0896-6273(93)90319-M 7679915

[19] Kam JWK Raja R Cloutier JF. Cellular and molecular mechanisms regulating embryonic neurogenesis in the rodent olfactory epithelium. Int J Dev Neurosci. (2014) 37:76–86. 10.1016/j.ijdevneu.2014.06.017 25003986

[20] Getchell ML Boggess MA Pruden II SJ Little SS Buch S Thomas V. Expression of TGF-β type II receptors in the olfactory epithelium and their regulation in TGF-α transgenic mice. Brain Res. (2002) 945:232–41. 10.1016/S0006-8993(02)02805-6 12126885

[21] Lei ZN Wu ZX Dong S Yang DH Zhang L Ke Z. Chloroquine and hydroxychloroquine in the treatment of malaria and repurposing in treating COVID-19. Pharmacol Ther. (2020) 216:107672. 10.1016/j.pharmthera.2020.107672 32910933 PMC7476892

[22] Fiolet T Guihur A Rebeaud ME Mulot M Peiffer-Smadja N Mahamat-Saleh Y. Effect of hydroxychloroquine with or without azithromycin on the mortality of coronavirus disease 2019 (COVID-19) patients: a systematic review and meta-analysis. Clin Microbiol Infect. (2020) 27:19–27. 10.1016/j.cmi.2020.08.022 32860962 PMC7449662

[23] Beigel JH Tomashek KM Dodd LE Mehta AK Zingman BS Kalil AC. Remdesivir for the treatment of Covid-19 - final report. N Engl J Med. (2020) 383:1813–26. 10.1056/NEJMoa2007764 32445440 PMC7262788

[24] Yonesi M Rezazadeh A. Plants as a prospective source of natural anti-viral compounds and oral vaccines against COVID-19 coronavirus. Preprints. (2020) 10.20944/preprints202004.0321.v1

[25] Ahn JY Kim MH Lim MJ Park S Lee S Yun YS. The inhibitory effect of ginsan on TGF-β mediated fibrotic process. J Cell Physiol. (2011) 226:1241–47. 10.1002/jcp.22452 20945375

[26] Ji X Cao J Zhang L Zhang Z Shuai W Yin W. Kaempferol protects renal fibrosis through activating the BMP-7-Smad1/5 signaling pathway. Biol Pharm Bull. (2020) 43:533–39. 10.1248/bpb.b19-01010 32115512

[27] Park SH Gong JH YJChoi Kang MK Kim YH Kang YH. Kaempferol inhibits endoplasmic reticulum stress-associated mucus hypersecretion in airway epithelial cells and ovalbumin-sensitized mice. PLoS ONE. (2015) 10:e0143526. 10.1371/journal.pone.0143526 26599511 PMC4657928

[28] Yang LT Liu X Cheng DY Fang X Mu M Hu XB. Effects of diterpene phenol extract of Rosmarinus officinalis on TGF-β1 and mRNA expressions of its signaling pathway molecules in the lung tissue of pulmonary fibrosis rats. Chin J Integr Tradit West Med. (2013) 33:819–24. 23980366

[29] Meurer SK Lahme B Tihaa L Weiskirchen R Gressner AM. N-acetyl-l-cysteine suppresses TGF-b signaling at distinct molecular steps: the biological efficacy of a multifunctional, antifibrotic drug. Biochem Pharmacol. (2005) 70:1026–34. 10.1016/j.bcp.2005.07.001 16098950

[30] Fu W Chen Y Wang K Hettinghouse A Hu W Wang JQ. Repurposing FDA-approved drugs for SARS-CoV-2 through an ELISA-based screening for the inhibition of RBD/ACE2 interaction. Protein Cell. (2020) 18:1–6. 10.1007/s13238-020-00803-w 33210243 PMC7673315

